# Comparison of scope holding sign on endoscopy and lower esophageal sphincter contraction on high‐resolution manometry: A pilot study

**DOI:** 10.1002/deo2.50

**Published:** 2021-09-20

**Authors:** Yusuke Fujiyoshi, Haruhiro Inoue, Yuto Shimamura, Mary Raina Angeli Fujiyoshi, Enrique Rodriguez de Santiago, Yohei Nishikawa, Akiko Toshimori, Mayo Tanabe, Kazuya Sumi, Yugo Iwaya, Masashi Ono, Shinya Izawa, Haruo Ikeda, Manabu Onimaru

**Affiliations:** ^1^ Digestive Diseases Center Showa University Koto Toyosu Hospital Tokyo Japan; ^2^ Department of Gastroenterology and Hepatology Hospital Universitario Ramón y Cajal, Universidad de Alcalá Madrid Spain; ^3^ Instituto Ramón y Cajal de Investigación Sanitaria (IRYCIS) Madrid Spain

**Keywords:** gastroesophageal reflux, lower esophageal sphincter, manometry

## Abstract

**Objectives:**

Lower esophageal sphincter (LES) plays a key role in gastroesophageal reflux disease (GERD) pathogenesis. In retroflexion and under sufficient insufflation, it can be seen how the lower esophagus grasps the endoscope, which we named scope holding sign (SHS). This study aimed to compare the SHS and LES pressure on high‐resolution manometry (HRM), to elucidate whether the sphincter can be visualized endoscopically.

**Methods:**

This was a single‐center, prospective pilot study. Patients with symptoms of GERD, who underwent endoscopy and HRM between February 2021 and April 2021, were included. A manometry catheter and an ultra‐slim endoscope were inserted, and the resting LES pressure was measured. The lower esophagus holding (SHS‐positive) and releasing (SHS‐negative) the endoscope and catheter were observed. The LES pressures during SHS‐positive and SHS‐negative were compared.

**Results:**

Eleven patients (median age: 57 years; eight men) with normal esophageal motility were analyzed. The median LES pressure in SHS‐positive was significantly higher than the resting LES pressure (40.4 [22.9–74.0] vs. 25.9 [2.0–66.7] mm Hg; *p* = 0.001) and the LES pressure in SHS‐negative (4.6 [1.5–9.3]; *p* = 0.001). Furthermore, the LES pressure in SHS‐negative was significantly lower than the resting LES pressure (4.6 [1.5–9.3] vs. 25.9 [2.0–66.7] mm Hg; *p* = 0.005).

**Conclusions:**

This study demonstrated that the SHS parallels LES pressure, indicating that the sphincter can be observed endoscopically. This may enable us to evaluate LES function during endoscopy in patients with GERD, thus, deserving further evaluation in future studies.

## INTRODUCTION

**FIGURE 1:**
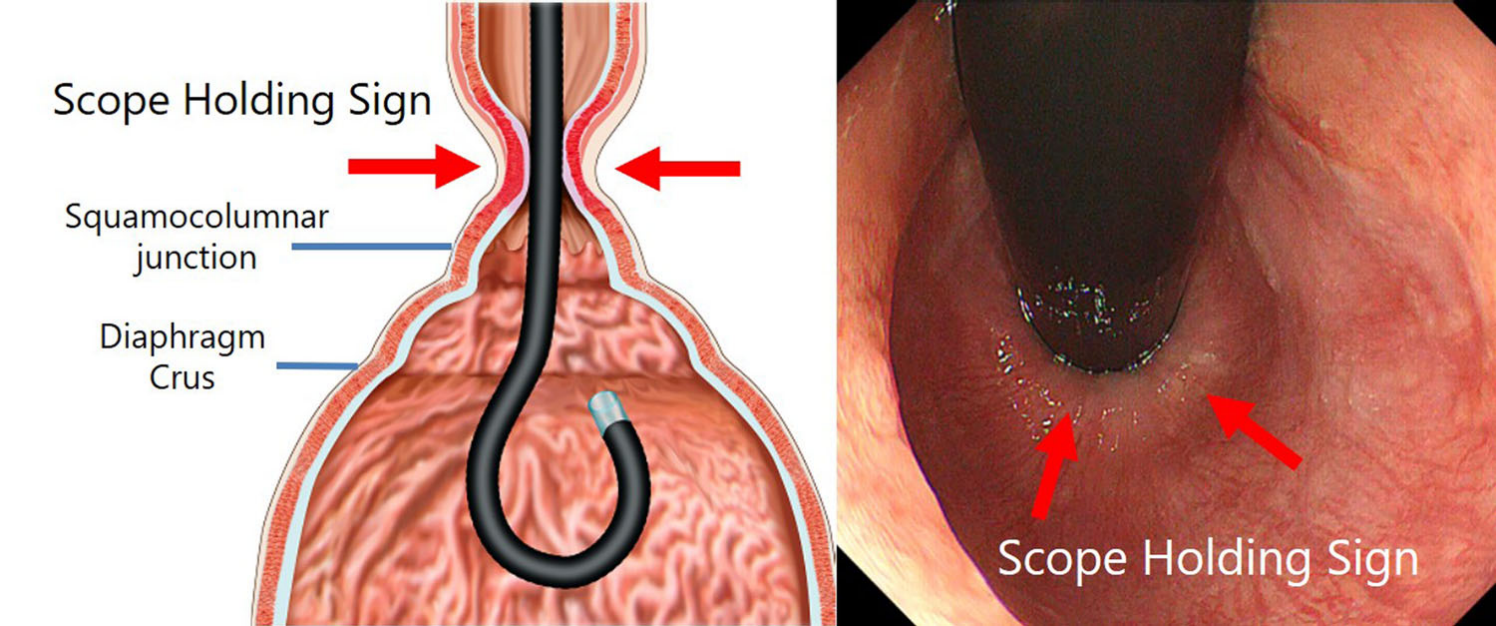
In the retroflexed view under sufficient insufflation, the lower esophagus holding the endoscope can be seen. This is the scope holding sign

Gastroesophageal reflux disease (GERD) is a frequent gastrointestinal disorder characterized by the presence of symptoms or complications resulting from the retrograde flow of gastric contents into the esophagus.^1^ The pathophysiology of GERD involves various mechanisms, but the lower esophageal sphincter (LES) plays an important role in the anti‐reflux function of the esophagus. The LES serves as a valve that prevents continuous acid reflux, and acid reflux occurs mainly during transient LES relaxation (TLESR).^2^ Normally, LES cannot be observed by endoscopy during forward view.^3^ However, on retroflexion and sufficient insufflation, it can be seen how the lower esophagus grasps the endoscope. We named this phenomenon (the lower esophagus holding the endoscope) the “scope holding sign” (SHS)^4^ (Figure 1). We hypothesized that LES contraction could be triggered by insufflation and observed as SHS using endoscopy. However, LES can be only identified by high‐resolution manometry (HRM), and SHS can be only observed by endoscopy. Therefore, in order to prove that LES can be observed endoscopically, this study aimed to compare the relationship between the SHS on endoscopy and LES contraction on HRM by simultaneously inserting the endoscope and HRM catheter. To our knowledge, this is the first study to report the correlation between endoscopic findings and LES contraction on high‐resolution manometry (HRM).

## METHODS

### Study population

This was a single‐center, observational, prospective pilot study. We included patients with GERD symptoms (heartburn, chest pain, or belching) but no significant sliding hiatal hernia (≤2 cm), who underwent upper gastrointestinal endoscopy and HRM with a unisensor catheter (Starlet, Starmedical Inc., Tokyo, Japan) between February 2021 and April 2021. Patients with major abnormalities in esophageal motility, such as absent contractility or minor abnormalities such as ineffective esophageal motility (IEM), were excluded. Absent contractility was defined as normal median integrated relaxation pressure and 100% failed peristalsis (distal contractile integral [DCI] <500 mm Hg·s·cm). IEM was defined as ≥50% ineffective swallows, which was due to a failed or weak peristalsis (DCI < 1000 mm Hg·s·cm).^5^, ^6^

### Procedure

An HRM system with a unisensor catheter (Starlet, Starmedical Inc., Tokyo, Japan) was used. Patients were placed in the supine position, and the catheter was inserted transnasally after placing lidocaine HCl 2% jelly in the nasal cavity. The position of the catheter was confirmed using deep inspirations. A baseline period of 30 s was captured to identify anatomic landmarks, including the LES, and the resting LES pressure was measured. The mean value of the resting LES pressure for 30 s was recorded. An ultra‐slim endoscope (GIF‐XP290N; Olympus Corporation, Tokyo, Japan; 5.8 mm in diameter) was inserted orally without removing the HRM catheter. The stomach was insufflated sufficiently with CO_2_, and on a retroflexed view, the lower esophagus holding the endoscope and HRM catheter (SHS‐positive) was observed, and the LES pressure was simultaneously measured. Sufficient insufflation is a state in which the folds of the greater curvature of the stomach flatten. On continuous CO_2_ insufflation, the lower esophagus releasing the endoscope and HRM catheter (SHS‐negative) was observed, and the LES pressure was simultaneously measured. The LES pressure in SHS‐positive was recorded as the mean value of the LES pressure during the whole duration when SHS is observed with endoscopy. Similarly, the LES pressure in SHS‐negative was recorded as the mean value of the LES pressure during the whole duration when SHS is not observed with endoscopy. As a reference, the intragastric pressure in SHS‐positive and negative was also recorded. To maintain the reproducibility of this method, the measurements were done twice, and the data of the second procedure were used. All patients were sedated with intravenous propofol (1%/20 ml) during endoscopy. The resting LES pressure, LES pressure during SHS‐positive and SHS‐negative were compared. Due to the nature of inserting HRM catheter and endoscope simultaneously, the HRM results where the catheter and endoscope interfered with each other were eliminated from the analysis.

### Statistical analysis

The median and range were calculated for continuous variables and frequency counts and percentages for categorical data. The Friedman test and post hoc Wilcoxon signed‐rank test were used to compare nonparametric paired continuous data of the three groups (resting LES pressure, LES pressure in SHS‐positive, and LES pressure in SHS‐negative). All analyses were two‐tailed. The significance after Bonferroni adjustment was 0.017, which was the result of dividing 0.05 by the number of comparisons (0.05/3 = 0.017). Therefore, *p*‐values less than 0.017 were considered significant in this study. All statistical analyses were conducted using the JMP 14 software (SAS Institute Inc., Cary, NC, USA) and STATA 16.1 software (Stata Corp, College Station, TX, USA).

### Ethical considerations

The study protocol adhered to the principles of the Declaration of Helsinki and was approved by the Institutional Review Board (IRB) of Showa University Koto Toyosu Hospital, Japan (IRB Registration No: 20T5023). Written informed consent was obtained from all participants.

## RESULTS

A total of 12 patients were screened. One case with a diagnosis of IEM did not show SHS and was excluded from the analysis. Thus, 11 patients who presented with normal esophageal motility on HRM were analyzed. The median (range) age was 57 (22–68) years, and eight patients were men (72.7%). Patient characteristics are presented in Table 1.

**TABLE 1:** Patient characteristics

Variable median (range)	(*n* = 11)
Age, years	58 (35–66)
Male gender	8 (72.7%)
BMI^†^, kg/m^2^	21.2 (16.2–29.0)
Sliding hernia, cm	1 (0–2)
Los Angeles classification (esophagitis)	
Grade N	9 (75%)
Grade A	2 (16.7%)
Grade B	1 (8.3%)
Grade C	0 (0%)
Grade D	0(0%)

^†^ Body mass index.

SHS was observed in all cases with sufficient insufflation. When the lower esophagus was holding the endoscope and HRM catheter (SHS‐positive), a high LES pressure was recorded on HRM. The *p*‐value of Friedman test for comparison of the three groups was *p* < 0.001 (resting LES pressure vs. LES pressure in SHS‐positive vs. LES pressure in SHS‐negative). The median LES pressure during SHS‐positive was significantly higher than the resting LES pressure (40.4 [22.9–74.0] vs. 25.9 [2.0–66.7] mm Hg; *p* = 0.001). When the lower esophagus released the endoscope and HRM catheter (SHS‐negative), the LES pressure decreased on HRM. The median LES pressure during SHS‐negative was significantly lower than the resting LES pressure (4.6 [1.5–9.3] vs. 25.9 [2.0–66.7] mm Hg; *p* = 0.005). Likewise, the median LES pressure during SHS‐positive was significantly higher than that during SHS‐negative (40.4 [22.9–74.0] vs. 4.6 [1.5–9.3]; *p* = 0.001; Figure 3). The results of each case, including resting LES pressure, LES pressure during SHS‐positive and SHS‐negative, are presented in Table 2.

**TABLE 2:** Cases in this study

Case number	Age (years)	Gender	BMI^†^ (kg/m^2^)	Gerd‐Q questionnaire	Esophagitis (LA^§^ grade)	Hill Grade	Sliding hernia (cm)	HRM^‡^ diagnosis	Resting LES^||^ pressure (mm Hg)	LES^||^ pressure in SHS¶ positive (mm Hg)	Intragastric pressure in SHS¶ positive (mm Hg)	LES^||^ pressure in SHS¶ negative (mm Hg)	Intragastric pressure in SHS¶ negative (mm Hg)
1	59	M	21.1	8	M	I	0	Normal	37.8	51.5	18.4	6.4	2.9
2	62	M	20.9	13	M	III	2	Normal	2.0	22.9	5.6	4.6	0.0
3	68	M	17.7	9	M	I	1	Normal	26.1	33.0	14.5	9.3	3.5
4	36	F	18.5	10	M	I	0	Normal	30.8	41.8	11.7	3.6	3.6
5	57	M	21.8	8	A	I	1	Normal	19.0	28.6	15.0	4.1	1.1
6	66	M	21.3	9	M	II	1	Normal	26.8	44.6	11.8	2.3	0.0
7	39	M	29.0	8	B	III	1	Normal	25.7	47.2	5.7	4.0	1.3
8	22	F	16.2	9	M	II	0	Normal	66.7	74.0	23.1	6.3	3.4
9	35	F	17.8	13	A	II	1	Normal	11.7	36.0	15.3	8.2	5.4
10	32	M	25.8	12	M	I	0	Normal	9.7	40.4	7.4	1.5	1.5
11	66	M	22.1	3	M	III	1	Normal	27.7	40.2	9.4	8.2	1.1

Abbreviations: F, female; M, male; N/A, not applicable.

^†^ Body mass index.

^‡^ High‐resolution manometry.

^§^ Los Angeles classification.

^||^ Lower esophageal sphincter.

^¶^ Scope holding sign.

## DISCUSSION

**FIGURE 2:**
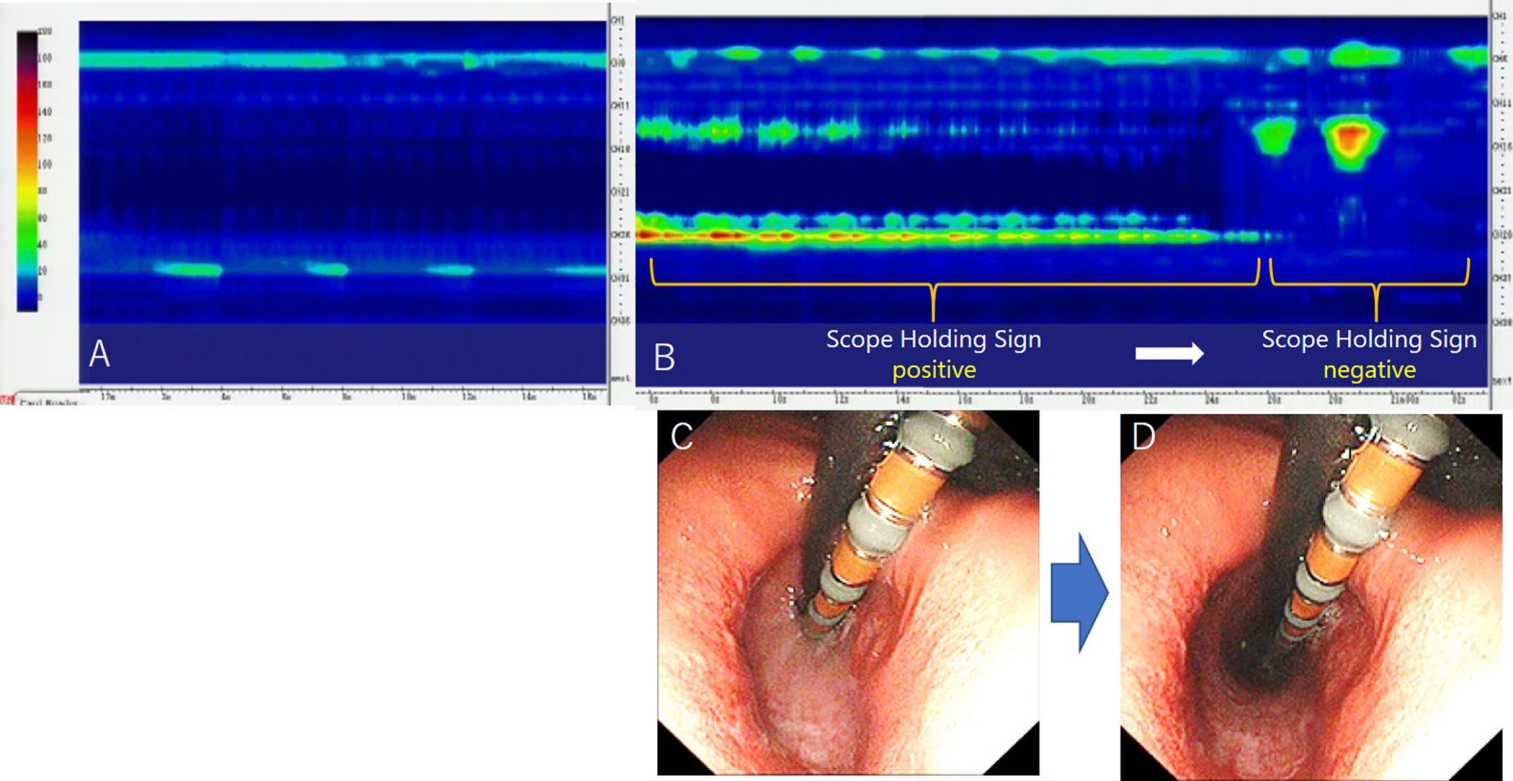
(a) HRM image of resting situation without the insertion of an ultra‐thin endoscope. (b) HRM image with the insertion of an ultra‐thin endoscope. (c) The lower esophagus holding the endoscope and HRM catheter (SHS‐positive); a high LES pressure is observed on HRM. (d) The lower esophagus releasing the endoscope and HRM catheter (SHS‐negative); the LES pressure decreased on HRM Abbreviations: HRM, high‐resolution manometry; LES: lower esophageal sphincter; SHS: scope holding sign.

In the present study, we prospectively assessed whether LES can be seen endoscopically during retroflexion by simultaneously inserting an ultra‐slim endoscope and HRM catheter. SHS is defined as the lower esophagus holding the endoscope on retroflexion and sufficient insufflation.^4^ The SHS on endoscopy coincided with the high LES pressure on HRM. In other words, the LES pressure during SHS‐positive was significantly higher than the resting LES pressure, while the LES pressure during SHS‐negative was significantly lower. To our knowledge, this is the first study to report the correlation between endoscopic findings and LES contraction on HRM. A significant increase in LES pressure compared to that in the resting situation was observed (Figure 2). These phenomena were triggered during CO_2_ insufflation, suggesting that continuous insufflation can cause LES contraction. Effects of acute intragastric pressure increase on LES have already been previously reported, and based on these findings, it was shown that abdominal compression^7^, ^8^, ^9^, ^10^ or compression by waist belt^11^ increased the intragastric pressure, thereby causing an increase in LES pressure. Another study showed that LES pressure subsequently increased with air insufflation into the stomach.^12^ In the present study, by the same mechanism, insufflation into the stomach caused the increase in the intragastric pressure and simultaneously, LES pressure increased, and SHS was observed. The results of the present study coincide with the previous reports.

**FIGURE 3:**
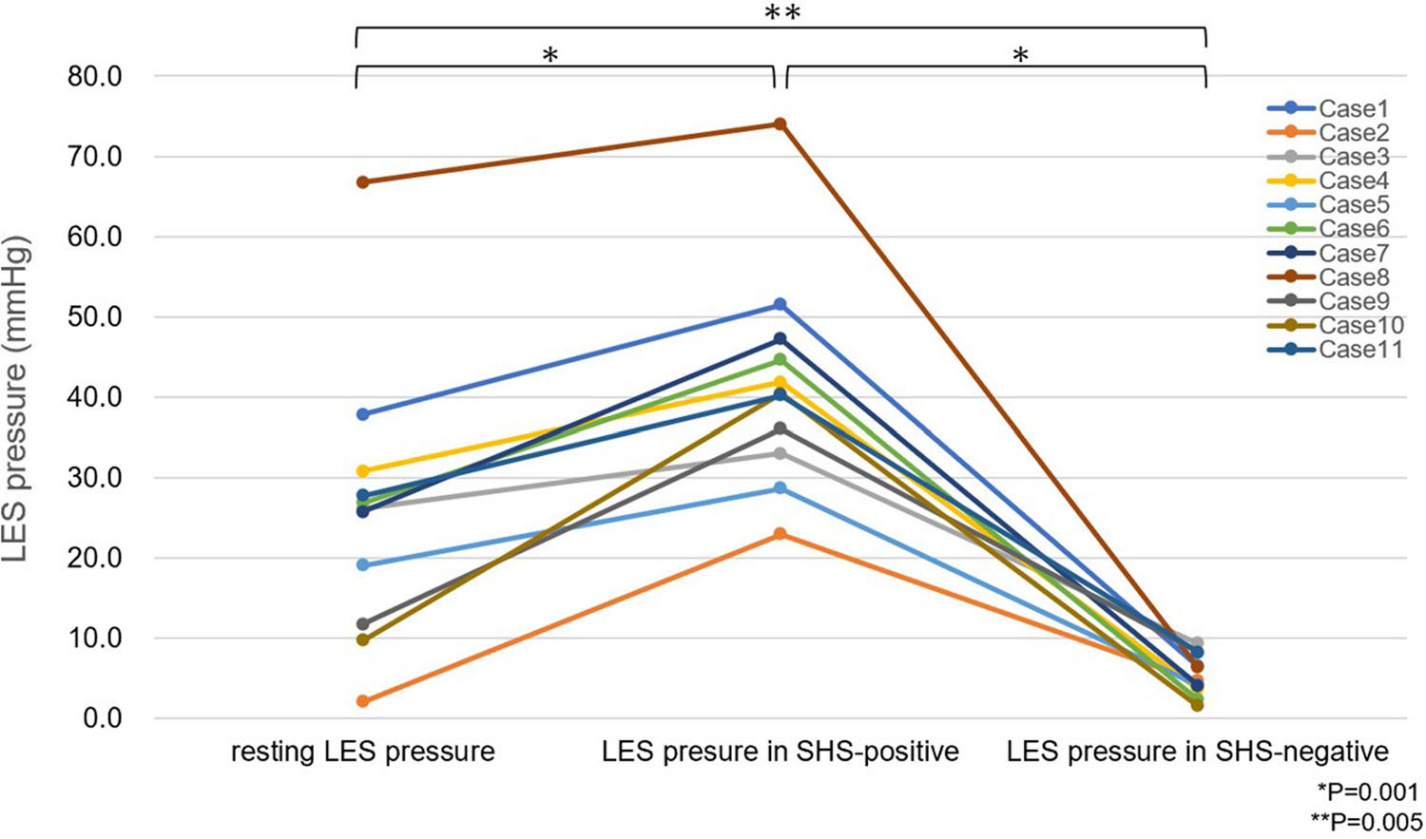
LES pressure in resting, SHS‐positive, and SHS‐negative conditions in each case Abbreviations: LES, lower esophageal sphincter; SHS, scope holding sign.

In addition, CO_2_ insufflation contributes to the displacement of the LES toward the proximal side (Figure 2). This coincides with a previous report stating that the gastroesophageal junction moves toward the proximal side with air insufflation into the stomach.^13^ Furthermore, it has been reported that gastric distention by air insufflation causes TLESR.^14^ Thus, we hypothesize that the phenomenon in which SHS‐positive turns negative may reflect TLESR. By applying our method of simultaneously inserting the endoscope and HRM catheter, the contraction and relaxation of the LES were observed endoscopically.

We have previously reported the usefulness of scope holding time percent (SHT%), which is the percentage of time wherein SHS is positive in 30 s.^4^ In a previous study, patients with low SHT% presented a significantly higher number of reflux episodes, and SHT% was considered to be a good marker for diagnosing GERD on endoscopy. Since the current study proved that SHS coincides with LES, it can be said that SHS and SHT% are the direct visualizations of LES using endoscopy. Endoscopically visualizing LES seems to be a novel approach, which may help in diagnosing and stratifying GERD patients.

Furthermore, we have previously reported a novel diagnostic tool for GERD, known as endoscopic pressure study integrated system (EPSIS).^15^, ^16^, ^17^ EPSIS allows evaluation of the anti‐reflux barrier during endoscopy by monitoring the intragastric pressure during stomach insufflation until the cardia opens and belching occurs. Considering that LES is the main anti‐reflux barrier, and belching occurs when the LES relaxes,^2^ we hypothesize that opening of the cardia before belching during EPSIS corresponds with the phenomenon wherein the SHS‐positive turns negative, implying that EPSIS evaluates TLESR. By utilizing this method of simultaneously inserting the HRM catheter and endoscope, the phenomenon during EPSIS in terms of LES function could be elucidated.

On a separate note, in the excluded patient, HRM findings showed IEM. IEM is defined as ≥50% ineffective swallows, which is due to a failed or weak peristalsis (DCI <1000 mm Hg·s·cm)^5^, ^6^ and is frequently observed in GERD patients.^18^ In the IEM case in this study, upon endoscopic examination with a retroflexed view, neither esophageal peristalsis nor high LES pressure was observed despite sufficient insufflation. This phenomenon suggests that patients with IEM might have decreased LES function as an anti‐reflux barrier of the esophagus. In future studies, we would like to evaluate the anti‐reflux function of the esophagus in cases of IEM.

This study has some limitations. First, this was a pilot study with a small sample size. Further studies to validate SHS and compare the SHS on endoscopy and LES pressure on HRM are warranted. Second, due to the simultaneous insertion of the endoscope and HRM catheter, the endoscope and HRM catheter may interfere with each other, causing the increase in pressure on HRM at the site of contact with the endoscope. In addition, this interference may result in not being zero in LES pressure even during LES opening (SHS‐negative). Moreover, due to the simultaneous insertion of the endoscope and HRM catheter, esophageal peristalsis may have been triggered by these foreign objects, which may have modified the HRM imaging results. These are the limitations of this method itself. Third, the sedation of all patients with propofol when performing HRM and endoscopy may have influenced the degree of relaxation of LES, thereby affecting the results of this study. Finally, we have not examined healthy individuals or severe reflux esophagitis patients in this study. In future studies, comparing LES pressures and SHS between healthy individuals and patients with GERD, and between patients with mild and severe reflux esophagitis is expected.

Overall, our pilot study demonstrated that SHS on endoscopy coincides with the highest LES pressure on HRM, indicating that SHS on endoscopy corresponds with LES contraction. This method may enable us to endoscopically evaluate LES function in patients with GERD and warrants further studies.

## CONFLICT OF INTEREST

Haruhiro Inoue is an advisor of Olympus Corporation and Top Corporation. He also received educational grants from Olympus Corporation and Takeda Pharmaceutical Company. Yusuke Fujiyoshi, Yuto Shimamura, Mary Raina Angeli Fujiyoshi, Enrique Rodriguez de Santiago, Yohei Nishikawa, Akiko Toshimori, Mayo Tanabe, Kazuya Sumi, Yugo Iwaya, Masashi Ono, Shinya Izawa, Haruo Ikeda, and Manabu Onimaru have no conflict of interests to declare.

## FUNDING INFORMATION

None.
